# Transcriptome Analysis Reveals Potential Mechanisms of L-Serine Production by *Escherichia coli* Fermentation in Different Carbon–Nitrogen Ratio Medium

**DOI:** 10.3390/foods11142092

**Published:** 2022-07-14

**Authors:** Zheng Chen, Xiaojia Chen, Qinyu Li, Peng Zhou, Zhijun Zhao, Baoguo Li

**Affiliations:** 1 School of Health Science and Engineering, University of Shanghai for Science and Technology, Shanghai 200093, China; 201570146@st.usst.edu.cn (Z.C.); 211310154@st.usst.edu.cn (P.Z.); 2 Lab of Biorefinery, Shanghai Advanced Research Institute, Chinese Academy of Sciences, Shanghai 201210, China; chenxj@shanghaitech.edu.cn (X.C.); liqy@shanghaitech.edu.cn (Q.L.)

**Keywords:** *E. coli*, transcriptome analysis, L-serine, fermentation

## Abstract

L-serine is an industrially valuable amino acid that is widely used in the food, cosmetics and pharmaceutical industries. In this study, transcriptome sequencing technology was applied to analyze the changes in gene expression levels during the synthesis of L-serine in *Escherichia coli* fermentation. The optimal carbon–nitrogen ratio for L-serine synthesis in *E. coli* was determined by setting five carbon–nitrogen ratios for shake flask fermentation. Transcriptome sequencing was performed on *E. coli* fermented in five carbon–nitrogen ratio medium in which a total of 791 differentially expressed genes (DEGs) were identified in the CZ4_vs_CZ1 group, including 212 upregulated genes and 579 downregulated genes. Gene Ontology (GO) and Kyoto Encyclopedia of Genes and Genomes (KEGG) enrichment analysis of these DEGs showed that the effect of an altered carbon–nitrogen ratio on the fermentability of *E. coli* was mainly focused on metabolic pathways such as GABAergic synapse and the two-component system (TCS) in which the genes playing key roles were mainly *gadB*, *gadA*, *glsA*, *glnA*, *narH* and *narJ*. In summary, these potential key metabolic pathways and key genes were proposed to provide valuable information for improving glucose conversion during *E. coli* fermentation.

## 1. Introduction

L-serine is generally considered a non-essential amino acid, and with the deepening of research, it was found that some vertebrates are unable to meet their cellular requirements with their synthesis of L-serine [1,2]. L-serine plays an important role in physiological metabolism and is closely related to the synthesis of compounds such as one-carbon units and purines [3]. Defects in L-serine biosynthesis may lead to developmental disorders, as well as neurological damage that cannot be completely cured, even with L-serine supplementation [4]. Recently, the role of L-serine in the treatment of Alzheimer’s disease was also identified. Cognitive deficits in mice with Alzheimer’s disease are closely related to impaired L-serine synthesis, which can be treated via oral administration of L-serine [5].

The traditional methods for the industrial production of L-serine are mainly protein hydrolysis and enzymatic conversion, which make it difficult to obtain further development due to their high cost and low yield [6]. Therefore, the method of obtaining L-serine via direct fermentation using cheap carbohydrate raw materials, such as glucose and sucrose, is gaining attention [7,8]. Among them, the technology of obtaining high-L-serine-yielding engineered *E. coli* through random mutagenesis and metabolic engineering is gradually being developed. After reasonable stoichiometric modeling, the theoretical yield of L-serine can reach 23.04 mmol/10 mmol glucose when *E. coli* consumes glucose to reach metabolic equilibrium, but the conversion of glucose in actual fermentation experiments is very low [9]. L-serine exists as an intermediate metabolite in the metabolic activity of *E. coli* and cannot be accumulated excessively. The synthesis process of L-serine is mainly regulated by four enzymes: 3-phosphoglycerate kinase, 3-phosphoglycerate dehydrogenase, phosphoserine aminotransferase and phosphoserine phosphatase (encoded by *pgk*, *serA*, *serC* and *serB*, respectively). The synthesis of L-serine is strictly feedback-inhibited, and the *serA* gene plays a key role in feedback inhibition. The main metabolic destination of L-serine is its conversion to glycine or pyruvate. The metabolic pathway of L-serine to pyruvate is mainly regulated by three L-serine deaminases (encoded by *sdaA*, *sdaB* and *tdcG*), while the metabolic pathway of L-serine to glycine is mainly regulated by a glycine hydroxymethyltransferase (SHMT, encoded by *glyA*) [10,11]. In the study by Rennig et al., the yield of L-serine reached up to 50 g/L with a glucose conversion rate of 0.36 g/g [12]. However, in a study by Mundhada et al. (2016), the yield of L-serine reached 0.43 g/g of glucose conversion at only 11.7 g/L [13]. Therefore, further improvement of glucose conversion is still of great significance for the industrial development of L-serine production via the *E. coli* fermentation of glucose.

The metabolic pathway of glucose in *E. coli* is very complex, and it is of great significance to clarify the molecular mechanism of the metabolic process and identify the key metabolic pathways or regulatory genes in order to improve the utilization of glucose. The development of transcriptome techniques has provided reliable information for revealing the expression of key metabolic pathways or key genes during growth metabolism [14,15,16]. Zhang et al. used glycerol and glucose as the only carbon sources for *Aurantiochyrium* to produce docosahexaenoic acid (DHA), and the results showed that glycerol was beneficial in that it increased the production of DHA. Transcriptome analysis revealed that glycerol promotes the expression of the tricarboxylic acid transport system and the polyketide synthase pathway, while glucose promotes the fatty acid synthesis pathway [17]. Lv et al. analyzed the fermentation performance of *Chlorococcum* sp. GD by setting up medium with different concentrations of glucose. High concentrations of glucose had negative effects on both biomass and photosynthesis. Transcriptome data revealed that high concentrations of glucose inhibited the expression of the citrate cycle (TCA cycle), oxidative phosphorylation and photosynthesis, thereby adversely affecting the growth of the microalga [18]. Zhang et al. investigated the effect of zinc sulfate addition on the production of β-glucan during a batch culture of *Aureobasidium pullulans* and analyzed it with the help of transcriptome sequencing technology. The results showed that the addition of zinc sulfate upregulated the expression of genes related to glucan synthesis and nucleotide metabolism, thereby increasing the yield of β-glucan [19]. Hirasawa et al. investigated the mechanism of penicillin-induced glutamate synthesis in *Corynebacterium glutamicum*. Transcriptomic results showed that transcript levels of genes involved in glycolysis and tricarboxylic acid cycle, as well as glutamate efflux, were upregulated and that the upregulation of expression levels of these genes benefited cellular metabolism by promoting glutamate production and transport to the extracellular compartment [20]. In addition, transcriptome sequencing was applied to reveal key metabolic pathways and genes in related studies on flavonoid biosynthesis in *Yarrowia lipolytica* tolerance, higher alcohol productivity of yeast and epidermal lupeol biosynthesis in *Ricinus communis* [21,22,23].

In the current research on L-serine production via fermentation in *E. coli*, the focus is still on the overexpression of genes related to L-serine synthesis (*pgk*, *serA*, *serC*, *serB*, etc.) or the deletion of genes related to the regulation of L-serine degradation (*sdaA*, *sdaB*, *tdcG*, *glyA*, etc.). However, more studies on the metabolic pathways or genes that regulate L-serine synthesis have rarely been reported. In this study, we used an engineered *E. coli* strain as the starting strain and designed the medium containing different carbon–nitrogen nutrient ratios for fermentation. There are potential differences in the expression of metabolic pathways or genes associated with L-serine synthesis in *E. coli* during fermentation. Transcriptome sequencing was used to reveal the enrichment and expression of differentially expressed genes (DEGs) in the fermentation process of the bacteria, thus identifying more key metabolic pathways and genes that regulate L-serine synthesis, and providing a research idea for the construction of engineered *E. coli* that synthesize L-serine more efficiently from carbon sources.

## 2. Materials and Methods

### 2.1. Bacteria, Plasmids and Medium

The *E. coli* used in this study were all derived from *E. coli* that are capable of producing the L-serine preserved in our laboratory. It was constructed from wild-type *E. coli* W3110 with both *sdaA* and *glyA* genes deleted, and was introduced into the low-copy number plasmid pSC. Plasmid pSC is a temperature-sensitive plasmid containing the temperature-sensitive repressor cItS857 gene and PR and PL promoters. The plasmid pSC contains the feedback-insensitive *serAfr* (H334A, D346A) gene, *serC* gene, *serB* gene and *pgk* gene.

Luria–Bertani (LB) medium: 10 g/L tryptone, 10 g/L NaCl, 5 g/L yeast extract, 15 g/L agar (solid medium).

Shake flask fermentation medium: 9 g/L glucose, 2 g/L yeast extract, 6.8 g/L NaHPO_4_, 3 g/L KH_2_PO_4_, 0.5 g/L NaCl, 0.49 g/L MgSO_4_•7H_2_O, 0.015 g/L CaCl_2_•2H_2_O, 2.8 × 10^−4^ FeSO_4_•7H_2_O, 1 mL/L 1000 × mother liquor of a composite additive of trace elements (1.5 g/L Na_2_MoO_4_•2H_2_O, 2.5 g/L CuSO_4_•5H_2_O, 3 g/L ZnSO_4_•7H_2_O, 7 g/L CoCl_2_•6H_2_O, 16 g/L MnCl_2_•4H_2_O, 25 g/L H_3_BO_3_) and 1 mL/L 1000 × vitamin B_1_ and vitamin H mother liquor (0.3 g/L vitamin B_1_, 4.5 g/L vitamin H); NH_4_Cl was calculated as 13.76, 6.88, 4.59, 2.75 and 1.38 g/L according to C/N = 1, 2, 3, 5 and 10, respectively. The C/N ≈ 2.6 of L-serine itself. In order to discover the optimal carbon–nitrogen ratio for the synthesis of L-serine and to combine the research of other researchers, C/N = 1, 2, 3, 5 and 10 were set [24]. The carbon-nitrogen ratio in the medium was calculated as the main carbon and the nitrogen sources were glucose and NH_4_Cl. Considering that the carbon–nitrogen ratio of L-serine itself is not an integer and the influence of other components in the medium, only the main carbon and nitrogen sources were calculated in this study.

### 2.2. Determination of Bacterial Growth

We referred to the method of Wang et al. [10] to determine the bacterial concentration. After turning on the UV-Vis spectrophotometer (DU730, Beckman, Krefeld, Germany), it needed to warm up for 20 min, and then the wavelength for analysis was selected. The bacterial concentration was determined by measuring the optical density at 600 nm. The bacterial concentrations were measured at 3, 5, 7, 9, 11, 13 and 15 h. The glucose content and L-serine content were measured at the same points as the bacterial concentration.

### 2.3. Determination of Glucose Content

The glucose content in the fermentation broth was determined using an SBA biosensor machine (Institute of Microbiology, Qingdao, China). It was first calibrated with glucose standard solution and then a 25 μL sample was aspirated for the determination [25].

### 2.4. Shake Flask Fermentation

*E. coli* plate-streaking experiments were performed on an LB solid medium and cultured in a constant-temperature incubator at 33 °C for about 24 h. The strain was activated by picking a single colony and incubating it for about 12 h in a tube with 3 mL of LB liquid medium at 33 °C and 200 rpm. Activation was performed three times, with each transfer involving 1% of the volume of the bacterial solution. The activated bacterial broth was inoculated into conical flasks containing 50 mL of shake flask fermentation medium at an 8% transfer volume for shake flask fermentation, incubation temperature 33 °C and speed 200 rpm. After 3 h of incubation, the temperature was increased to 38 °C to induce the production of L-serine by the bacteria [10].

### 2.5. Determination of L-Serine Content

The fermentation broth samples were centrifuged at 12,000 r/min for 10 min and the supernatant was subjected to pre-column derivatization by referring to the method of Chen et al. [26]. HPLC analysis was subsequently performed using a Shimadzu RID-10A/SPD-20A, Japan. Referring to the method of Wang et al., the analytical conditions were as follows [10]:

Agilent Extend C-18 column (250 mm × 4.6 mm, 5 µm). Mobile phase A was 0.05 mol/L sodium acetate (pH 6.50 ± 0.05). Mobile phase B was methanol/acetonitrile/water (20:60:20, *v*/*v*/*v*. Gradient elution (0~11 min: 85% mobile phase A + 15% mobile phase B, 11~15 min: 0 mobile phase A+100% mobile phase B, 15~25 min: 15% mobile phase A + 15% mobile phase B). Flow rate 0.8 mL/min. The UV detection wavelength was 256 nm and the injection volume was 10 μL.

### 2.6. Transcriptome Sequencing

#### 2.6.1. RNA Extraction and Library Construction

Transcriptome sequencing was performed on the bacterial broth grown to the logarithmic phase during the shake flask fermentation process. The bacterial broth was snap-frozen in liquid nitrogen for storage and total RNA was extracted from the broth using the Total RNA Extractor (Trizol) extraction kit according to the manufacturer’s method. The RNA concentration, integrity and genomic contamination were examined using Qubit 2.0 and agarose gels to ensure the smooth execution of subsequent experiments. The library construction was performed using the VAHTS™ Stranded mRNA-seq Library Prep Kit for Illumina^®^ according to the manufacturer’s methods.

#### 2.6.2. RNA Sequencing and DEGs Analysis

Sangon Biotech (Shanghai, China) Co., Ltd., completed the transcriptome sequencing. Raw reads were obtained by sequencing on the Illumina Hiseq™ platform, and then the raw reads were processed using Trimmomatic to remove low-quality reads and poly-N reads, and thus, obtain clean reads. Genome mapping analysis of clean reads and reference genomes was performed using Bowtie2. DESeq was used for the analysis of DEGs, and the screening condition for significantly different genes was q-value ≤ 0.05 and |log2FoldChange| ≥ 1.

#### 2.6.3. Gene Ontology (GO) and Kyoto Encyclopedia of Genes and Genomes (KEGG) Enrichment Analysis

The topGO was used for GO enrichment analysis. The clusterProfiler was used for KEGG enrichment analysis, and the screening condition for functionally significant enrichment was a corrected p-value (q-value) < 0.05.

### 2.7. Data Analysis

The experimental data of this study were expressed as mean ± standard deviation. Origin 9.1 software was used to plot the study pictures. SPSS 24 software was used for the data analysis.

## 3. Results and Discussion

### 3.1. Effect of Carbon–Nitrogen Ratio Differences in Culture Medium on the Fermentation Performance of Shake Flasks

**Figure 1:**
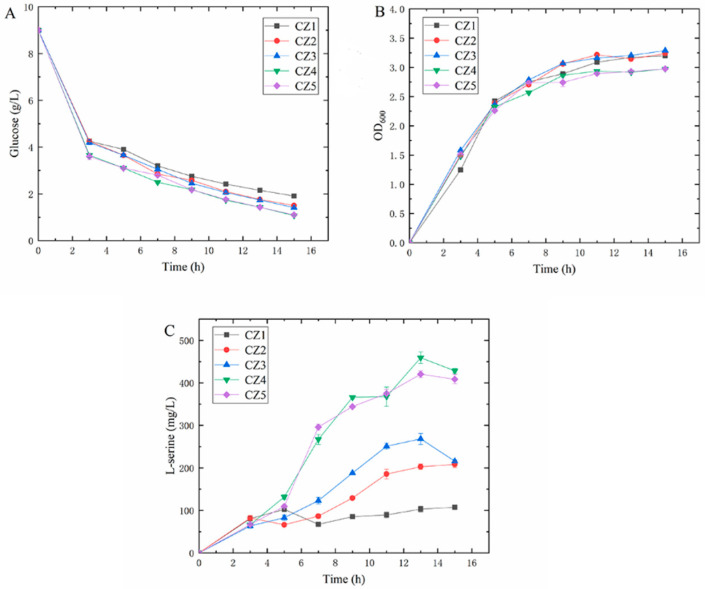
Experimental results of shake flask fermentation with different carbon–nitrogen ratios. (**A**) Glucose residue in the fermentation broth; (**B**) bacterial accumulation in the fermentation broth; (**C**) L-serine production in the fermentation broth.

In order to investigate the effect of the differences in carbon–nitrogen ratio in the culture medium on the ability of *E. coli* fermentation to produce L-serine, we set up five different culture medium components with C/N = 1, 2, 3, 5 and 10, which were named the CZ1, CZ2, CZ3, CZ4 and CZ5 groups, respectively. The results of the shake flask fermentation experiment are shown in Figure 1. The glucose content in the fermentation broth was depleted faster during the first 3 h of high-temperature-induced plasmid expression, which is when the bacteria grew faster and required large consumption of glucose. After the induction of plasmid expression due to the elevated temperature, the rate of glucose consumption decreased, at which time the bacteria began to accelerate the accumulation of L-serine. The induction temperature was more favorable for the growth of *E. coli*; however, the growth rate of *E. coli* started to decrease. This result may be related to the insufficient supply of glucose in the fermentation broth to offset the promotional effect of the suitable temperature on the growth of *E. coli* [27]. By the end of fermentation, the CZ4 group had the highest glucose consumption and the residual glucose in the fermentation broth was 1.07 g/L. The residual glucose in the CZ5 group was 1.10 g/L. From the growth status of the bacteria, it was found that by the end of the fermentation, the accumulation of the bacteria in the CZ4 and CZ5 groups was also the lowest, and the OD_600_ values were both 2.98. From the L-serine production, it was found that the L-serine production of the CZ4 and CZ5 groups was significantly higher than the other three groups during the whole fermentation process. In particular, the CZ4 group reached the highest L-serine yield of 428.42 mg/L by 13 h of fermentation. Changes in the composition of the medium may have an impact on a strain’s metabolic pathways, and the status of the synthesis of the target product is subsequently altered [28]. It can be seen that the carbon–nitrogen ratio of 5 was the most favorable for *E. coli* to synthesize L-serine, and the consumption capacity of glucose was also stronger than the other experimental groups, while the accumulation of bacterial concentration was lower than the other experimental groups. This result may have been related to the accumulation of L-serine in the fermentation broth, which interfered with the synthesis of branched-chain amino acids in bacteria, thereby inhibiting the growth of bacteria [29]. It was also found that the yield of L-serine in this study was still far from the theoretical yield; therefore, it is necessary to explore new methods to improve the conversion of glucose to L-serine.

### 3.2. Transcriptome Analysis

#### 3.2.1. Quality Control of Sequencing Data for Transcriptome Analysis

For the shake flask fermentation experiments with different carbon–nitrogen ratios, 28,472,982, 66,408,726, 32,292,894, 31,288,250 and 30,827,104 raw reads were obtained from the CZ1, CZ2, CZ3, CZ4 and CZ5 groups, respectively. Raw reads obtained using transcriptome sequencing have a certain error rate, which has a negative impact on the later bioinformatics analysis. Therefore, the raw reads are processed using Trimmomatic to remove low-quality reads and poly-N reads, and information about the resulting clean reads is shown in Table 1. The number of all clean reads in the samples of five groups ranged from 27,860,276 to 65,073,878, with more than 96.03% of base masses above 30 and more than a 52.47% GC content. Therefore, the data obtained in the early stage were highly reliable and could be used for the subsequent analysis.

#### 3.2.2. Reference Sequence Comparison Analysis

Clean reads were filtered using sequencing data and then mapped on the reference sequence for analysis, and the results are shown in Table 2. The clean reads that could be mapped to the reference genomes in the CZ1, CZ2, CZ3, CZ4 and CZ5 groups reached 27,433,999, 63,563,985, 31,033,431, 30,085,238 and 29,597,379, respectively, with the percentages ranging from 97.93% to 98.52%. Among them, the percentages of clean reads with multiple mapped positions on the reference genome ranged from 2.67% to 12.98%, while the percentages of clean reads with only unique mapped positions ranged from 84.95% to 95.63%. This result indicated that the sequencing samples were not contaminated and the results were highly reliable.

**Figure 2:**
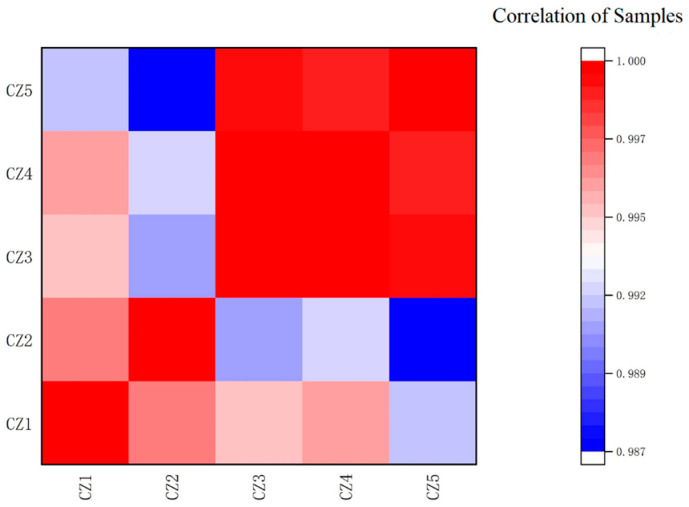
Heat map of correlation analysis between samples.

The correlation of the gene expression levels between samples is represented by the Pearson correlation coefficient. The closer the correlation coefficient is to 1, the more similar the gene expression patterns of the samples are. As shown in Figure 2, the Pearson correlation coefficients between different samples ranged from 0.9865 to 0.9999, which indicated that the correlation of gene expression levels between the samples was high and further justified the sample selection.

#### 3.2.3. Screening of DEGs

**Figure 3:**
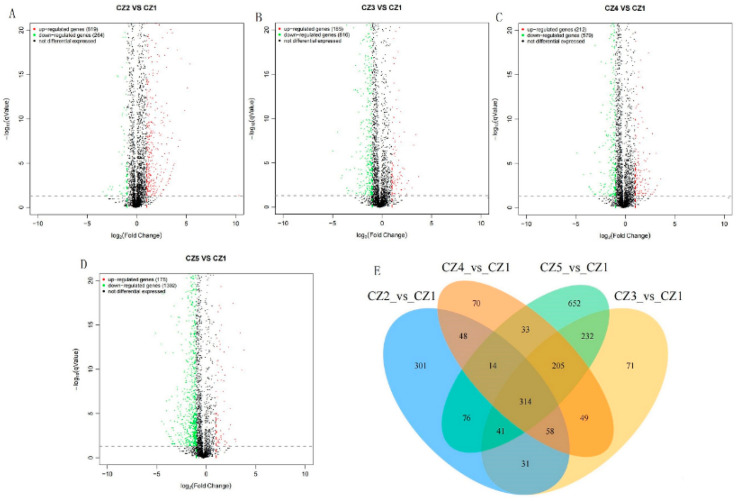
Analysis of differentially expressed genes (DEGs). (**A**–**D**) The horizontal axis indicates the differential expression multiplicity fold-change (log (B/A)) value of the gene, while the vertical axis indicates the statistical significance of the change in gene expression p-value. The smaller the p-value, the larger the −log (p-value), and the more significant the difference. (**E**) The numbers in the graph indicate the number of genes that are specific or common between the comparison groups.

In order to explore the differential genes that affect the fermentation performance of *E. coli* in different carbon–nitrogen ratio medium, the CZ1 group samples were analyzed for differences with the CZ2, CZ3, CZ4 and CZ5 group samples, and the results are shown in Figure 3. Compared with the CZ1 group, a total of 883 DEGs were screened in the CZ2 group, of which 619 genes were upregulated and 264 genes were downregulated. Compared with the CZ1 group, a total of 1001 DEGs were screened in the CZ3 group, of which 185 genes were upregulated and 816 genes were downregulated. Compared with the CZ1 group, a total of 791 DEGs were screened in the CZ4 group, of which 212 genes were upregulated and 579 genes were downregulated. Compared with the CZ1 group, a total of 1567 DEGs were screened in the CZ5 group, of which 175 genes were upregulated and 1392 genes were downregulated. The comparison of these four groups in differential genes also revealed that 314 of these differential genes were present in all groups of comparisons. This result indicated that the carbon–nitrogen ratio caused a large change in the gene expression level of *E. coli*. Different carbon–nitrogen ratios in the medium will result in corresponding changes in gene expression, that is, DEGs. Differences in fermentation performance are the result of differences in DEGs. Therefore, these DEGs are of great significance for improving the production level of L-serine.

#### 3.2.4. GO Enrichment Analysis of DEGs

**Figure 4:**
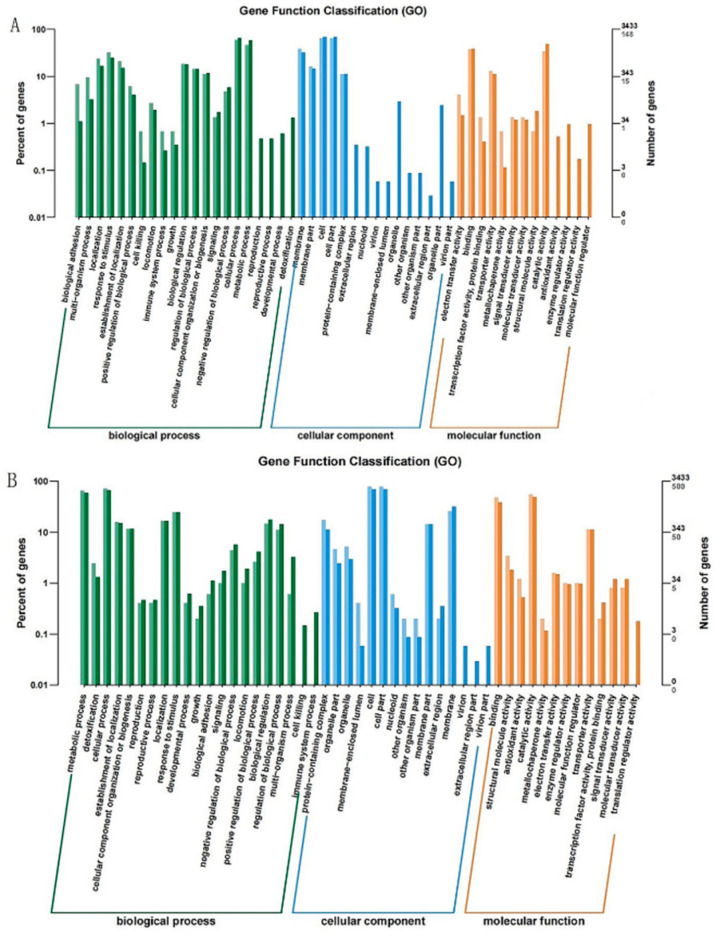
Gene Ontology (GO) enrichment analysis of DEGs. (**A**) Upregulated genes analysis results; (**B**) downregulated genes analysis results.

It was known from the shake flask fermentation experiment that the CZ4 group had the highest L-serine production; therefore, this study focused on analyzing the DEGs in the CZ4_vs_CZ1 group. The DEGs in the CZ4_vs_CZ1 group included 212 upregulated genes and 579 downregulated genes. GO enrichment analysis was performed for these two groups separately, and the results are shown in Figure 4. The results of GO enrichment analysis mainly included 21 functional units involved in biological processes, 15 functional units in cellular locations and 13 functional units involved in molecular functions. The upregulated genes in the CZ4 group were mainly enriched in biological processes, such as localization, response to stimulus, the establishment of localization, biological regulation, regulation of biological processes, cellular component organization or biogenesis, cellular processes and metabolic processes. The molecular functions performed by upregulated genes mainly included binding, transporter activity and catalytic activity, and mainly function in membrane and protein-containing cellular sites, such as the membrane, membrane part, cell, cell part and protein-containing complex. The downregulated genes in the CZ4 group were mainly enriched in biological processes such as metabolic processes, cellular processes, the establishment of localization, cellular component organization or biogenesis, localization, response to stimulus, negative regulation of biological processes, biological regulation and regulation of biological processes. Its molecular functions mainly included binding, structural molecule activity, catalytic activity and transporter activity, and the position of action is also mainly concentrated in the cell position containing membrane and protein, such as the protein-containing complex, organelle part, organelle, cell, cell part, membrane part and membrane. From this result, it can be seen that the significantly upregulated or downregulated genes involved in the functional expression were involved in similar situations. It was also found that by changing the carbon–nitrogen ratio of the medium, the activity of proteases on the cells could be significantly influenced, which in turn regulated the growth and metabolite production of the cells.

#### 3.2.5. KEGG Metabolic Pathway Analysis of DEGs

**Figure 5:**
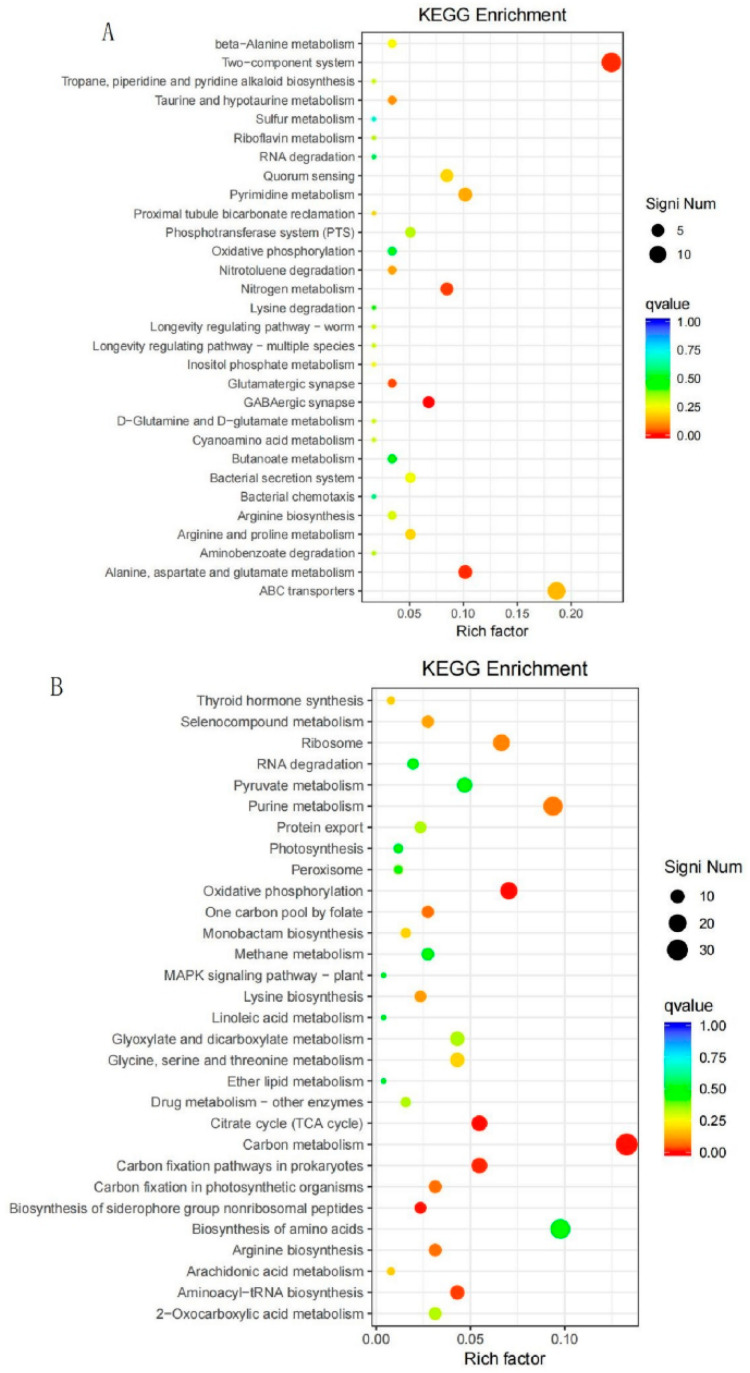
Scatter plot of DEGs Kyoto Encyclopedia of Genes and Genomes (KEGG) enrichment function. (**A**) Upregulated genes analysis results; (**B**) downregulated genes analysis results.

KEGG metabolic pathway enrichment analysis was performed on the DEGs of the CZ4_vs_CZ1 group, and a total of 315 significantly DEGs were annotated using KEGG and involved in 110 metabolic pathways. Among them, there were 59 significantly upregulated DEGs involved in 27 metabolic pathways and 256 significantly downregulated DEGs involved in 104 metabolic pathways. The 30 metabolic pathways with the highest enrichment were selected for analysis and the results are shown in Figure 5. Among the most enriched metabolic pathways, those that were significantly upregulated were focused on the two-component system (TCS); nitrogen metabolism; glutamatergic synapse; GABAergic synapse; and alanine, aspartate and glutamate metabolism. Meanwhile, those that were significantly down-regulated were focused on oxidative phosphorylation, one carbon pool by folate, arginine biosynthesis, carbon fixation in photosynthetic organisms, purine metabolism, the TCA cycle, carbon metabolism, carbon fixation pathways in prokaryotes, biosynthesis of siderophore group nonribosomal peptides and aminoacyl−tRNA biosynthesis.

### 3.3. Mining of Key Metabolic Pathways and Genes for L-Serine Production in E. coli

Comparing the GO and KEGG enrichment analysis results of the CZ5_vs_CZ1 group, CZ4_vs_CZ1 group, CZ3_vs_CZ1 group and CZ2_vs_CZ1 group, it was found that both the GABAergic synapse and TCS were significantly enriched with a large number of DEGs, which had a significant effect on the fermentability of *E. coli*. The analysis results of the effect of these two metabolic pathways on the fermentability of *E. coli* are given below using the CZ4_vs_CZ1 group as an example.

#### 3.3.1. GABAergic Synapse

GABA is a non-protein amino acid widely found in various microorganisms and plays an important role in message transmission as an inhibitory neurotransmitter [30,31]. In glutamate metabolism, glutamate undergoes decarboxylation to form GABA, and *gadB* and *gadA* are the only rate-limiting enzymes in this reaction [32]. In the GABAergic synapse metabolic pathway of *E. coli*, the significantly DEGs were mainly involved in the synthesis of GABA. The gene *gadB* was upregulated by 1.36 times, the gene *gadA* was upregulated by 1.42 times, the gene *glsA* was upregulated by 1.25 times and the gene *glnA* was upregulated by 2.2 times. The increased transcription levels of the genes *gadB*, *gadA* and *glsA* promoted the synthesis of GABA, while the gene *glnA* mainly regulated the conversion of glutamate to glutamine in glial cells, and the generated glutamine was then transferred and involved in the glutamate metabolism system. Serine is a key phosphorylation site of the GABA receptor, and the accumulation of serine can promote the function of GABA [33]. The accumulation of L-serine promoted the glutamate metabolic system to synthesize more GABA to act on the TCA cycle, and the growth of the bacteria was inhibited because GABA could inhibit the TCA cycle to provide material and energy for the growth of the bacteria. Therefore, from the comparison of the CZ4_vs_CZ1 group, it was found that the increase in the carbon–nitrogen ratio promoted the synthesis of L-serine while inhibiting the growth of bacteria.

#### 3.3.2. TCS

The TCS is a signal transduction system that exists in a wide range of bacteria and which can sense and respond to changes in the external environment with time. A typical TCS mainly consists of two parts: histidine kinase (HK) and response regulator (RR). HK responds to environmental signals through autophosphorylation, and the phosphoryl groups generated by phosphorylation are transferred to RR to cause cellular physiological changes, which are usually achieved by altering the gene expression [34,35,36]. As observed from the transcriptome sequencing results of the CZ4 group, significant upregulation of the genes *glnL*, *glnG* and *glnA* promoted the expression of the glutamate metabolic system functions. It was shown that the ability of *E. coli* to resist acidic environments is dependent on L-glutamine. L-glutamine can be converted to L-glutamate via the action of glutaminase under the stimulation of acid. At the same time, ammonia gas is released into the environment, and the released ammonia causes the pH of the environment to rise, achieving the effect of an anti-acid [37,38]. During the synthesis of L-serine by *E. coli*, the pH decreased with the utilization of glucose. The TCS metabolic pathway can maintain the pH of the fermentation broth, which is beneficial to the stable synthesis of L-serine by *E. coli*. The upregulation of the *narH* and *narJ* gene transcript levels promotes the expression of nitrogen metabolic systems. In the nitrogen metabolic pathway of *E. coli*, extracellular nitrate is transported into the cell via the nitrate/nitrite transport system substrate-binding proteins. Subsequent conversion to nitrite under the regulation of genes, such as *narH*, or direct extracellular transport into the cell, with the resulting nitrate eventually being used to synthesize ammonia to participate in the glutamate metabolic pathway [39]. It can be seen that the TCS is closely related to nitrogen metabolism and glutamate metabolism, and there are common significantly different genes involved in these metabolic pathways.

## 4. Conclusions

In this study, the shake flask fermentation experiment confirmed that a carbon–nitrogen ratio of 5 was beneficial for the fermentation of *E. coli* to produce L-serine, and the maximum yield of L-serine reached 428.42 mg/L under this condition. The improvement in the synthesis efficiency of glucose to L-serine during the fermentation process is beneficial to reduce the industrial production cost of L-serine. Therefore, transcriptome sequencing technology was applied to mine metabolic pathways and genes that are closely related to glucose utilization during *E. coli* fermentation. The comprehensive analysis of the transcriptome sequencing results from the CZ5_vs_CZ1, CZ4_vs_CZ1, CZ3_vs_CZ1 and CZ2_vs_CZ1 groups showed that both the GABAergic synapse and TCS metabolic pathways enriched a large number of significantly DEGs (*gadB*, *gadA*, *glsA*, *glnA*, *narH* and *narJ*). These key metabolic pathways promote L-serine synthesis while also improving the adaptation of *E. coli* to adverse environments. Therefore, the results of this research will provide valuable information for the construction of engineered *E. coli* that synthesize L-serine more efficiently from carbon sources.

## Figures and Tables

**Table 1:** Transcriptome data quality statistics.

Sample	Total Reads Count	Total Bases Count (bp)	Average Read Length (bp)	Q20 Bases Ratio (%)	Q30 Bases Ratio (%)	GC Bases Ratio (%)
CZ1	27,860,276	4,127,583,116	148.15	98.96	96.08	52.94
CZ2	65,073,878	9,459,591,029	145.37	99.02	96.31	52.76
CZ3	31,583,064	4,686,203,693	148.38	98.96	96.03	52.60
CZ4	30,706,250	4,560,411,727	148.52	99.01	96.16	52.63
CZ5	30,088,160	4,463,605,237	148.35	98.98	96.09	52.47

**Table 2:** Reference genome comparison results.

Sample	Total Reads	Total Mapped	Mutiple Mapped	Uniquely Mapped	Reads Mapped to ‘+’	Reads Mapped to ‘−’	Non-Spliced Reads	Reads Mapped in Proper Pairs
CZ1	27,844,844	27,433,999 (98.52%)	857,821 (3.08%)	26,576,178 (95.44%)	13,288,453	13,287,725	26,576,178	26,181,602
CZ2	64,908,882	63,563,985 (97.93%)	8,423,620 (12.98%)	55,140,365 (84.95%)	27,571,051	27,569,314	55,140,365	50,666,474
CZ3	31,569,790	31,033,431 (98.30%)	844,243 (2.67%)	30,189,188 (95.63%)	15,097,174	15,092,014	30,189,188	29,876,526
CZ4	30,683,164	30,085,238 (98.05%)	822,624 (2.68%)	29,262,614 (95.37%)	14,633,739	14,628,875	29,262,614	28,974,756
CZ5	30,064,744	29,597,379 (98.45%)	977,979 (3.25%)	28,619,400 (95.19%)	14,310,590	14,308,810	28,619,400	28,259,990

## Data Availability

No new data were created or analyzed in this study. Data sharing is not applicable to this article.
